# APOE Genotype, ApoE Plasma Levels, Lipid Metabolism, and Cognition in Monozygotic Twins with, at Risk of, and without Affective Disorders

**DOI:** 10.3390/jcm13082361

**Published:** 2024-04-18

**Authors:** Jon Dyg Sperling, Ruth Frikke-Schmidt, Thomas Scheike, Lars Vedel Kessing, Kamilla Miskowiak, Maj Vinberg

**Affiliations:** 1 The Early Multimodular Prevention and Intervention Research Institution (EMPIRI), Mental Health Centre, Northern Zealand, Copenhagen University Hospital—Mental Health Services CPH, 3400 North Zealand, Denmark; maj.vinberg@regionh.dk; 2 Department of Clinical Medicine, Faculty of Health and Medical Sciences, University of Copenhagen, 2200 Copenhagen, Denmarklars.vedel.kessing@regionh.dk (L.V.K.);; 3 Department of Clinical Biochemistry Rigshospitalet, Copenhagen University Hospital, 2100 Copenhagen, Denmark; 4 Department of Biostatistics, University of Copenhagen, 1353 Copenhagen, Denmark; 5 Copenhagen Affective Disorders Research Centre (CADIC), Psychiatric Center Copenhagen, 2000 Frederiksberg, Denmark; 6 Neurocognition and Emotion in Affective Disorders (NEAD) Centre, Department of Psychology, University of Copenhagen, and Mental Health Services, Capital Region of Denmark, 2000 Frederiksberg, Denmark

**Keywords:** affective disorders, cognition, monozygotic twins, high-risk-study, apolipoprotein E, lipid metabolism

## Abstract

**Background**: Lipids influence brain function and mental health. Understanding the role of apolipoproteins in affective disorders could provide valuable insights and potentially pave the way for novel therapeutic approaches. **Methods:** We examined the apolipoprotein E genotype and ApoE-levels, lipid profiles, and the correlation with cognition in 204 monozygotic (MZ) twins with unipolar or bipolar disorder in remission or partial remission (affected, AT), their unaffected co-twins (high-risk, HR), and twins with no personal or family history of affective disorder (low-risk, LR). **Results:** The APOE genotype was not associated with affective disorders. No significant group differences in ApoE levels were found between the three risk groups. Post hoc analysis group-wise comparisons showed higher ApoE levels in the AT than HR twins and in the concordant AT twin pairs relative to the discordant twin pairs. Within the discordant twin pairs, higher ApoE levels were observed in the affected twins (AT = 39.4 mg/L vs. HR = 36.8 mg/L, *p* = 0.037). **Limitations:** The present study could benefit from a larger sample size. We did not assess dietary habits. **Conclusions:** The results did not support our main hypothesis. However, exploratory post hoc analysis suggests a role for plasma ApoE and triglycerides in affective disorders. Future research is needed.

## 1. Introduction

Unipolar disorder (UD) affects approximately 280 million and bipolar disorder (BD) affects 50 million people globally [1], and affective disorders (uni- and bipolar disorders) are among the leading causes of disability worldwide [2]. The underlying pathophysiology is not well characterized, but the disorders are multifactorial, and evidence from twin, family, and adoption studies indicates a strong genetic predisposition [3,4].

A diverse plethora of risk and prediction markers have been investigated in mood disorders, such as early indicators in youth, substance abuse, family history, childhood life events [5,6,7]. Also, brain networks and neural makers have been explored in mood disorders [8,9] as well as the association between mood disorders and various health conditions. Studies have indicated a relationship between mood disorders and adverse outcomes like COVID-19 susceptibility, hospitalization, and death [10]

Further, affective disorders often co-occur with metabolic diseases such as cardiovascular diseases (CVDs), obesity, and non-insulin-dependent diabetes (NIDDM) [11,12,13]. Metabolic diseases also express a high heritability [14,15], which may include a shared genetic predisposition to affective disorders [16,17,18]. Affective disorders and metabolic diseases thus seem to share pathophysiological mechanisms that manifest as different conditions in different organs (i.e., heart and brain) [19]

Both patients with affective disorders and metabolic diseases may experience impaired cognitive function and have a higher risk of developing dementia, according to research [20,21,22]. Approximately 50% of patients with affective disorders have impaired cognitive function [23,24,25], including attention, verbal memory, and executive function, and their disorders are associated with overall impaired functioning [26]. Patients with NIDDM, overweight and obesity, and metabolic diseases express a similar cognitive pattern [27].

Lipid homeostasis plays a role in the development of neuropsychiatric disorders. As the brain is our most lipid-rich organ and apolipoproteins are responsible for transporting and metabolizing lipids, emerging evidence substantiates that these proteins may play a part in the critical health of the brain [28,29], implying that apolipoprotein proteins could be involved in brain disease development [30].

Adverse lipoprotein patterns have been found in patients with MDD [31]. Lipid and lipoprotein levels are influenced by the APOE polymorphism, which encompasses six common APOE genotypes, including E2, E3, and E4 alleles. Both E2 and E4 alleles are linked to adverse lipid profiles, with the E4 allele being a significant genetic risk factor for Alzheimer’s disease [32]. While an earlier study from our group found no difference in the frequency of the APOE4 allele between patients with affective disorders and healthy controls [33], recent meta-analyses have implicated APOE more broadly in severe mental disorders and affective disorders [11,34,35]. Apolipoprotein E plasma level (ApoE), the product of APOE, plays a crucial role in lipid metabolism and is produced primarily by the liver and macrophages in peripheral tissues, while astrocytes mainly produce ApoE in the central nervous system (CNS). In the brain, ApoE serves as the principal cholesterol carrier and is essential for the clearance of beta-amyloid plaques, the hallmark of Alzheimer’s disease [36]. Low plasma levels of ApoE have been associated with an increased risk of dementia, with higher levels linked to a higher risk of ischemic heart disease [37]. Although few studies have explored the impact of ApoE levels on affective disorders, one study [38] has investigated ApoE levels before and after medical treatment in patients with bipolar disorder, finding that patients not treated with psychotropics had lower ApoE and that it increased following treatment. Another study [39] has examined ApoA and ApoB levels in major depressive disorder (MDD), revealing that severity of depression correlated to higher ApoB and inversely to ApoA. Given the potential implications of the APOE genotypes in brain disorders and their association with lipid levels, further investigation into the role of lipids and ApoE in mood disorders is warranted.

Our group has previously revealed that monozygotic (MZ) twins with affective disorder and their discordant unaffected co-twins had a higher prevalence of metabolic syndrome than healthy MZ twins [40]. Based on the same sample of MZ twins, the present study aims to investigate APOE polymorphisms, ApoE-, and lipid levels across various risk levels for affective disorders and further possible correlations with cognition. We have hypothesized that the affected twins would express different distributions of APOE genotypes and higher ApoE and lipid levels than the low-risk twins, with the high-risk twins discordant for affective disorder expressing intermediary levels. We have further hypothesized that higher ApoE and lipid levels correlate with poorer cognition.

## 2. Materials and Methods

### 2.1. Design

Monozygotic twins were identified through a nationwide record linkage of The Danish Twin Registry (DTR), The Danish Psychiatric Central Research Centre (DPCRR), and The Danish Civil Registration System (for further details, see [41]. The record linkage identified MZ twins diagnosed with unipolar or bipolar disorder according to ICD-10 and DF30-39 criteria between 1995 and 2014, their unaffected high-risk co-twin, and a group of low-risk twin pairs. Recruitment took place between December 2014 and January 2017. Participants were invited by letter and further contacted by phone.

The MZ twins were classified into affected twins (in remission or partial remission), unaffected high-risk twins (co-twin affected), and low-risk twins without a personal or first-degree relative history of affective disorder. The twins were further divided into concordant pairs (both twins with affective disorder), discordant pairs (twin pairs with one affected twin and one unaffected high-risk twin), and healthy pairs, low-risk twins (psychiatrically healthy twin pairs; both twins have no personal history or first-degree relatives with schizophrenia or affective disorders). The twins who participated without their co-twin participating were classified according to the registry information and the psychiatric assessment.

Diagnoses were confirmed using the Schedules for Clinical Assessment in Neuropsychiatry (SCAN) interview [42], and affective symptoms were assessed using the Hamilton Depression Rating Scale (HDRS-17) [43] and the Young Mania Rating Scale (YMRS) [44] with remission or partial remission scored at ≤14. Two MD Ph.D. students blinded to the DPCRR-registered diagnoses performed ratings and interviews. Exclusion criteria were prior head trauma with unconsciousness and sequelae, birthweight > 1300 g, pregnancy, current substance abuse, severe somatic illness, and dizygotic twins.

### 2.2. Ethics

This study was approved by the Danish National Board of Health, the Data Protection Agency (2014-331-0751), and the regional ethical committee (H-3-2014-003). The project was completed in accordance with the Declaration of Helsinki, and all the participants gave informed written consent.

### 2.3. Measures

Blood samples were collected between 9 and 11 a.m. after 15 min of rest. Blood was immediately kept on ice and centrifuged within one hour for 15 min at 4 °C. Blood sampling and all aspects of the laboratory processing were performed at the Department of Clinical Biochemistry, Rigshospitalet, by laboratory specialists blinded to participant status. Lipid parameters were analyzed using Cobas 8000, c702 module (Roche, Basel, Switzerland). Total cholesterol, HDL cholesterol, and triglycerides were measured in mmol/L. LDL cholesterol was calculated as total cholesterol – HDL cholesterol – (0.45 × triglycerides) and given in mmol/L.

Plasma was stored in Eppendorf tubes at −80 degrees until analysis. ApoE was measured using nephelometry or turbidimetry (Dade Behring, Deerfield, IL, USA, or Dako, Glostrup, Denmark). For APOE genotyping, we used an ABI PRISM 7900HT Sequence Detection System (Applied Biosystems Inc., Foster City, CA, USA), and Taqman-based assays were used to genotype for p.Cys130Arg (rs429358, legacy name Cys112Arg, c.388T>C) defining the E4 allele, and for p.Arg176Cys (rs7412, legacy name Arg158Cys, c.526C>T) defining the E2 allele. The laboratory personnel were blinded to the participant’s diagnostic status, and the samples were randomly assigned across assays.

A participant-category-blinded assessor assessed cognitive performance. Processing speed and executive functioning, including working memory, were measured with the Trail Making Test A (TMT-A) and B (TMT-B) [45] and the Screen for Cognitive Impairment in Psychiatry (SCIP-D) [46]. The SCIP-D measures objective cognitive functioning and is comprised of five subtests measuring verbal learning and delayed memory (VLT-L, VLT-D), working memory (WMT), verbal fluency (VLF), and processing speed (PS). The SCIP instrument was validated in patients with affective disorders [46,47]. A higher SCIP score means better performance, while higher TMT-A and -B scores equal inferior performance.

### 2.4. Statistical Analyses

To investigate the association of polymorphism with the three categories, healthy twin pairs, discordant twin pairs, and concordant affected twin pairs, we used Fisher’s exact test. The six genotypes were analyzed in the following groups: APOE22; APOE32, APOE33 (reference category); APOE42; APOE43, and APOE44. The model considered the group a fixed factor/predictor variable. ApoE and lipid levels were analyzed as continuous dependent variables with group as a fixed factor by using a mixed model univariate analysis of variance (ANOVA) with the random factor taking intra-twin dependability (ITD) into account. All analyses were performed unadjusted and adjusted for age and gender.

For the continuous dependent variables, the analyses were three-fold: The primary analysis compared the three risk groups: affected twins (twins with affective disorder in complete or partial remission), high-risk twins (healthy twin with an affected co-twin), and low-risk twins (healthy twins with no personal or first-degree family history of affective disorder). The secondary analysis compared concordant affected twin pairs (both twins with affective disorders), the discordant twin pairs (one affected, one healthy), and the low-risk twin pairs to detect whether the concordant affected twin pairs would reveal poorer outcomes than the discordant twin-pairs. The tertiary analysis compared intra-pair differences between the affected and unaffected twins in the discordant twin pairs using a paired *t*-test. All associations and correlations were obtained exploratorily.

Exploratory correlation analyses between biomarkers and cognition were conducted using the Spearman correlation test. All analyses were performed using IBM SPSS Statis-tics Institute version 29.

Using IBM SPSS statistics, we conducted post hoc power calculations using our published data examining the specific biomarker, ‘Triglycerides’. With a sample size of *n* = 143 and a mean triglyceride difference of 0.34 (mean SD difference 0.411) μ/L, a power of 0.944 was achieved to identify a statistically significant difference between groups at a significance level of 0.05 (two-tailed). When comparing the high-risk (*n* = 40) and low-risk groups (*n* = 38), we achieved a power of 0.288 with a mean triglyceride difference of 0.121 μ/L (mean SD difference 0.03).

## 3. Results

**Figure 1:**
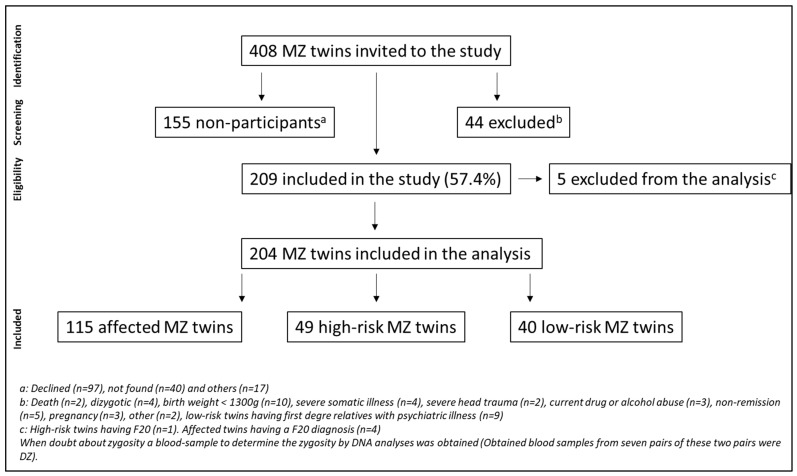
Flowchart of participant inclusion process. Monozygotic participants having an affective disorder (affected twins), a co-twin with affective disorder (high-risk twin), or no family history of affective disorder (low-risk twin).

As seen in Figure 1, 408 MZ twins were invited. These potential participants were found from all available MZ twins in the Danish Twin Registry at the time (age 18–50 years). Of them, 44 were excluded, and 155 declined to participate (n = 101) or could not be reached (n = 54). In total, 209 twins were seen for assessment, and five were excluded due to a personal or first-degree family history of schizophrenia or schizotypal disorder (details are described in [41]).

The analyses included 204 participants (Table 1): 115 MZ twins with affective disorder (73% unipolar disorder, 27% bipolar disorder, mean remission time 42.7 months), 49 at high risk, and 40 at low risk. The three risk groups were comparable in age, gender distribution, years of education, and alcohol consumption. The affected group were more often smokers (*p* = 0.038), less occupied (*p* = 0.001), and had higher HDRS17 scores (*p* = 0.008).

### 3.1. Apolipoprotein E Genotype Risk Mediation

**Figure 2:**
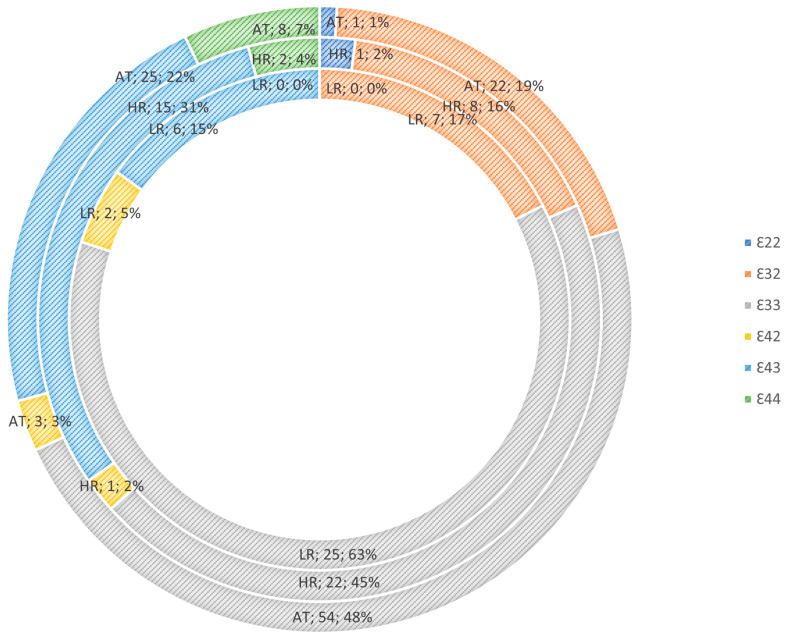
Apolipoprotein E (APOE) genotype distribution between the three monozygotic twin risk groups for affective disorders. Inner-circle: low-risk group, LR. Middle-circle: high-risk group, HR. Outer-circle: affected group, AT.

Table 2 shows the frequency distribution of the APOE genotype across concordant, discordant, and low-risk twin pairs. No statistically significant differences were found (*p* = 0.436) using Fisher’s exact test. Figure 2 shows the frequency distribution of the APOE genotype across risk groups and illustrates the ratios between the three risk groups. Table 2 and Figure 2 show that E44 homozygotes were only represented in the discordant twin pairs (n = 4, 4.4%) and in concordant affected twin pairs (n = 6, 11.6%), and none were seen in the LR twin pairs. E43 was more frequent in the discordant twin pairs (28.9%) than in the LR twin pairs (15.8%) and the concordant twin pairs (15.1%). Looking at the trend of the distribution ratios of the risk groups, Figure 2 illustrates that the affected group (AT) had the following genotype frequency: E44: 7%, E43: 25%; the HR group had E44: 4% and E43: 31%; and the LR group had E44: 0% and E43: 15.0%. Both unadjusted analyses and analyses adjusted for age and gender were carried out without changing the trend or statistical significance.

### 3.2. Biomarker Risk-Group Analysis

Table 3 shows the primary, secondary, and tertiary analysis comparing the affected *p* (n = 105), high-risk (n = 48), and low-risk groups’ (n = 38) plasma levels of ApoE, triglycerides, HDL, LDL, and cholesterol.

### 3.3. Apolipoprotein E Levels

The primary analysis revealed no statistically significant group differences in ApoE levels between the affected, high-risk, and low-risk groups. However, post hoc analyses in-between groups comparing the affected and the high-risk groups with the low-risk twins revealed statistically significant differences between the affected (41.9 mg/L) and the high-risk twins (38.9 mg/L), respectively, in unadjusted analyses (*p* = 0.035) and analyses adjusted for age and sex (*p* = 0.045).

The secondary analyses comparing the concordant affected pairs (n = 25) and the discordant twin pairs (n = 45) with the low-risk twin pairs (n = 19) showed no overall significant differences between the three groups. Post hoc pairwise group comparison revealed that the concordant affected twin pairs had statistically significantly higher ApoE levels (44.0 mg/L) than the discordant group (38.6 mg/L), both unadjusted (*p* = 0.046) and at a trend level when adjusted for age and sex (*p* = 0.054). The tertiary analyses comparing the affected and the unaffected twins in the 45 discordant twin pairs revealed that the affected twins had statistically higher ApoE levels than the discordant unaffected twins (39.4 mg/L vs. 36.8 mg/L, *p* = 0.037).

Finally, exploratory post hoc analyses were conducted to investigate the possible effect of the APOE genotypes. We found an overall significant effect of genotype on ApoE levels (*p* < 0.001), and the association increased the *p*-value when comparing the affected twins vs. the high-risk twins (*p* = 0.013).

### 3.4. Triglyceride Levels

Overall, in the primary analyses, a strong trend towards the affected twins having statistically significantly higher triglycerides than the low-risk twins (1.1 mmol/L, 95% CI:1.0–1.2 vs. 0.8 mmol/L, 95% CI:0.5–1.0, *p* = 0.052) was seen. Pairwise group comparison showed a difference between the affected and low-risk twins (*p* = 0.026) in the unadjusted analyses. Adjusted for age and sex, there were statistically significant differences between the high-risk twins vs. the affected twins (*p* = 0.012) and the high-risk twins vs. the low-risk twins (*p* = 0.048).

In the secondary analysis, triglyceride levels were statistically significantly higher when comparing the concordant affected pairs: 1.3 mmol/L with the discordant pairs: 0.9 mmol/L; low-risk pairs: 0.8 mmol/L (*p* = 0.025). Unadjusted pairwise group comparisons showed that the concordant affected twin pairs’ triglyceride levels were statistically significantly higher than low-risk twin pairs (1.3 mmol/L vs. 0.8 mmol/L, *p* = 0.003) and between the concordant affected pairs vs. the discordant twin pair (1.3 mmol/L vs. 0.9 mmol/L, *p* = 0.008). Finally, in the tertiary analyses, triglyceride levels did not differ between the affected and unaffected twins. Exploratory post hoc analyses were conducted to investigate the effect of adjusting for APOE genotypes, and no statistically significant effect of genotype on triglyceride levels was observed.

### 3.5. High Density Lipoprotein, Low-Density Lipoprotein, and Cholesterol Levels

When conducting primary, secondary, and tertiary analyses, no statistically significant results were found for HDL, LDL, and cholesterol both overall and as pairwise comparisons (results not presented in detail).

### 3.6. Post hoc Correlations between Cognition and ApoE and Lipid Levels

Table 4 presents Spearman’s correlation coefficient, including ApoE and lipid levels and cognition across all participants. First, ApoE and triglyceride levels showed no significant correlations with the cognitive measures. In contrast, higher cholesterol levels did show correlations with better working memory (*p* = 0.050, r = −0.14) and psychomotor speed (*p* = 0.026, r = −0.16), and poorer attention (TMT-A: *p* < 0.001, r = 0.21) and executive function (TMT-B: *p* = 0.01, r = 0.18). High-density lipoprotein showed statistically significant positive correlations with better verbal learning and memory (SCIP-VLT-I: *p* = 0.00, r = 0.24; VLT-D *p* = 0.02, r = 0.17), and with total SCIP scores (*p* = 0.02, r = 0.16).

Low-density lipoprotein correlated with poorer cognitive performance on the tests of psychomotor speed (*p* = 0.04, r = −0.14), attention (TMT-A: *p* = 0.02, r = 0.18), and executive function (TMT-B: *p* = 0.02, r = 0.17). Finally, ApoE levels were moderate positive correlated with triglycerides (*p* = 0.000, r = 0.44), cholesterol (*p* = 0.000, r = 0.41), and LDL (*p* = 0.000, r = 0.35).

## 4. Discussion

In contrast to our hypothesis, this study involving 204 MZ twins at various risks for affective disorder comparing affected, high-risk, and control twins, revealed differences in the distributions of the APOE genotypes. No significant group differences in ApoE levels were found between the three risk groups; however, within the discordant twin pairs, higher ApoE levels were observed in the AT than the HR twins (AT = 39.4 mg/L, HR = 36.8 mg/L, *p* = 0.037). Finally, the AT twins had significantly higher triglyceride levels than the LR twins (AT: 1.1 mmol/L, HR: 0.9 mmol/L, LR: 0.8 mmol/L, *p* = 0.026).

ApoE levels were further positively correlated with triglycerides, cholesterol, and LDL levels. Post hoc analyses showed that ApoE- and triglyceride levels did not correlate with cognitive performance, but ApoE levels correlated with an increased metabolic risk profile. There were weak correlations between higher lipid levels and poorer cognitive performance.

### 4.1. APOE Genotype

Contrary to our hypothesis, we did not find a significantly higher frequency of APOE44 or E43 in the concordant affected twin pairs compared to the discordant twin pairs and LR twin pairs. As the E4 allele is associated with disease state, we expected homozygotes of this allele, E44, to be more frequent among the affected twins. As can be seen from Figure 2, there was a trend in the distribution ratios of the risk groups E43 + 44: the concordant affected twin pair = 29.2%, the discordant twin pairs = 34.7%, and the LR twin pairs = 15%. This could indicate a more frequent representation of the E4 allele among the affected and the high-risk MZ twins than in the low-risk twins, but only at a trend level, which may be due to the modest sample size.

Several studies have highlighted the importance of the APOE genotype as a risk factor associated with CNS pathology [28,29,48]. A meta-analysis on the association between APOE genotype and depression found that in a Caucasian population, the E2 allele likely acted as a protective factor for depression, while the E44 and E43 genotypes acted as risk inducers [49]. This was also to some extent mirrored in an association study of APOE genotype and cognition in bipolar disorder [50], where the E2 allele presented improved cognitive performance while the presence of the E4 allele was associated with worse performance in some cognitive tasks. However, most studies were from Asia, so the included samples are ethnically different from the Danish population. Nevertheless, our study’s results are in line with the results from the previously mentioned study [33].

### 4.2. Apolipoprotein E Levels

This is the first study to report on plasma ApoE levels in a sample of MZ twins with, at risk for, and without affective disorders. While we found no overall differences in ApoE levels between the three risk groups, there were significant post hoc differences between the affected group vs. the high-risk group and between the concordant affected twin pairs vs. the discordant twin pairs. Finally, comparing the discordant MZ twin pairs, ApoE levels were higher in the affected twins.

The primary results are not in alignment with our hypotheses, as the high-risk group had the lowest ApoE levels while the affected and low-risk groups had higher or similar results. A possible explanation could be that low ApoE levels may be a result of a compensation factor. Affective disorders are also associated with an increased risk of dementia [51,52], and as lower ApoE levels may be associated with dementia [53], we expected that the affected twins would have decreased ApoE levels. However, lower levels may only be seen in the more severe stages of both affective disorders and dementia. Hence, the higher levels present in this study may be explained by the relatively young age of the affected twins, mean age = 36.1, and the fact that they had been in remission or partly remitted for a long time (mean 42.7 months). In contrast to our findings, Dean et al. [38] have reported diminished ApoE plasma levels in patients with bipolar disorder undergoing medical treatment compared to healthy controls. Notably, the use of ApoE plasma level ratios in their study precludes a precise commentary on the magnitude of the observed plasma level variations [38].

### 4.3. Triglyceride Levels

Having an affective disorder was associated with higher triglyceride levels. This may be a ‘scarring’ effect of the disorders. However, the intra-twin pair analysis of the discordant twin pairs does not support this assumption, as no statistically significant difference between the affected and the unaffected twin was observed. In the group-wise comparison of the risk groups, the high-risk twins also expressed significantly higher triglycerides levels than the low-risk twins (the adjusted post hoc analysis), which could point towards triglycerides being associated with a risk of affective disorders. This is in line with our previous finding of higher rates of metabolic syndrome in both the affected and the high-risk twins [40]. Triglycerides are a shared risk factor for dementia and atherosclerotic CVD [54], and they are also increased in affective disorder [55,56]. Thus, increased triglyceride levels may act as a risk factor for cognitive impairment, dementia, and ischemic stroke in patients with affective disorder.

### 4.4. Correlation between Biomarkers and Cognition

ApoE and triglyceride levels were not correlated with the cognitive measures. Cholesterol and LDL only showed a weak correlation (<0.2), with the cognitive measures pointing toward a proposition that higher levels (and lower levels of HDL) were correlated with poorer cognitive performance. However, due to the small sample size, these results warrant further investigation.

There are no previous reports on possible correlations between plasma ApoE and cognition in affective disorder. However, animal (mice) studies have shown that APOE ‘knock-out’ mice experience cognitive impairment, severe dyslipidemia, and atherosclerosis [57]. After the restoration of plasma ApoE to wild-type levels, both lipids and learning and memory difficulties fully normalized again, providing evidence for the proposition that ApoE levels are associated with cognitive function. Two studies examining the correlation/association between ApoE levels and cognition in elderly populations were identified; however, incongruence was observed between these investigations [58,59].

Higher triglyceride levels are suspected to be correlated with cognitive impairment. It has been shown in mice models that the administration of triglycerides to mice decreases learning and memory [60], and that elevated triglycerides are associated with depression and cognitive impairment in humans [61]. A recent study has found that parts of the metabolic syndrome cluster were significantly associated with cognitive impairment across psychiatric disorders and that the summative effects of individual components were the best predictor of cognition and the identification of individuals with worse outcomes [62]. An older meta-analysis [63] revealed only weak evidence to support the idea that cholesterol levels are associated with cognitive decline, which is in line with our findings. Finally, one study has revealed that individuals with lower LDL levels had a slower cognitive decline than individuals with higher LDL levels [64].

### 4.5. Strengths and Limitations

The MZ study design offers the possibility to stratify ultra-high-risk twins and affected twins. As MZ twins share nearly identical genes, forming better conclusions about endophenotypes is possible. Additionally, this study shows a comprehensive data collection of a large MZ twin sample which were recruited through nationwide register linkage, and the register-based recruitment reduced selection bias. However, several limitations should be considered. As this is a cross-sectional study, we cannot draw conclusions about causality. The study would have benefitted from a larger sample size and the sub-analysis should therefore be regarded as exploratory only.

Different potentially confounding variables may have influenced the analyses of both metabolism and cognition. For instance, lifestyle factors such as smoking differed between groups, as the affected and the high-risk group were more often smokers. Alcohol consumption did not differ between groups. We did not collect data on physical activity and dietary habits, which may have influenced the results. However, fine-grained dietary data are challenging to collect and are often influenced by substantial self-report bias, and lifestyle habits may impact metabolic syndrome [65].

MZ twins may not be representative of the background population; nevertheless, several studies have found that MZ twins, in comparison to singletons, do not differ with regard to frequency of cancer, diabetes, education, height, or bipolar disorder [66,67,68,69].

The present study only included a brief battery of cognitive tests; thus, the used cognitive tests may not have captured all of the cognitive domains sufficiently. The affected twins underwent neurocognitive testing during full or partial remission, rendering it possible that subsyndromal symptoms may have affected their cognitive test performance. Finally, the potential confounding effects that different medications or previous psychoses may have had on cognition may have influenced the results, as these factors affect cognition [70]. The participants were tested under a standardized setting at approximately the same time of day and were not allowed to drink coffee or smoke before the test. Benzodiazepines were tapered to a maximum of 22.5 mg oxazepam (or equivalent).

Future investigations into the relationship between lipid metabolism, APOE, cognition and mood disorders are needed to shed light on the field. One perspective is to conduct interventional studies to evaluate the effect of targeting metabolism through pharmacological treatments, e.g., statins, medicine with weight-lowering potential, or lifestyle interventions, to understand the interplay further. Investigating further genetic and epigenetic factors that may modulate metabolism is also warranted, e.g., target interventions in individuals at high metabolic risk. These could benefit from longitudinal studies, including advanced biomarker and brain imaging technologies.

### 4.6. Clinical Implications and Treatment Strategies

Affective disorders reduce lifetime expectancy by approximately eight years compared with the general population [71,72,73], and this is mainly due to lifestyle diseases and secondary suicide. Patients with affective disorders are at an increased risk of developing cardiovascular disease (CVD) and are affected on average ten years earlier than individuals without mental disorders [74,75]. Further, the prevalence of overweight and type-2 diabetes, respectively, are twice as frequent as in the general population [76]. Hence, lipid metabolism is of importance in affective disorders.

Here, we have observed triglycerides as a state marker of affective disorders in affected twins. These results point toward targeting metabolomics as a therapeutic augmentation target in mood disorders. Further, using the “usual suspects”, total cholesterol, high-density lipoprotein, low-density lipoprotein, and triglycerides, would be an advantage, as these biomarkers are already included in daily clinical settings.

## 5. Conclusions

We have found no association between APOE genotype and affective disorder in this sample of MZ twins at various risks of affective disorders. However, affective disorders were associated with higher ApoE levels, and ApoE seems to be associated with an increased metabolic risk profile. Metabolomic biomarkers only showed a weak correlation with cognitive measures. Overall, these findings align with the hypothesis that a proportion of patients with mood disorders seem to share common pathophysiological mechanisms with overweight, obesity, CVD, and NIDDM. However, the results warrant further investigation using longitudinal study designs.

## Figures and Tables

**Table 1:** Socio demographics, affective symptoms, smoking and alcohol use, cognition scores (means and standard deviation, SD), diagnoses, and medication in affected, high-risk, and low-risk monozygotic twins.

Risk Status	Affected	High Risk	Low Risk	*p*-Value
Number, n	115	49	40	
Demographics				
Age, years (SD)	36.1 (8.8)	36.9 (9.6)	37.1 (9.2)	0.868
Sex female, n (%)	75 (65.2)	34 (69.4)	24 (60.0)	0.652
Years of education, (SD)	14.5 (3.3)	15.7 (3.1)	15.3 (2.6)	0.123
In occupation, n (employment + education, %):	65 (57.0)	40 (81.6)	32 (80.0)	0.001
Smoking and alcohol				
Currently smoking, n (%)	35 (30.4)	13 (26.5)	4 (10.0)	0.038
Alcohol consumption (units/week, SD)	2.5 (4.0)	3.8 (5.3)	3.6 (3.1)	0.563
Affective symptoms				
HDRS-17 (SD)	4.4 (3.7)	4.1 (3.6)	2.2 (2.3)	0.008
YMRS (SD)	1.8 (2.1)	1.5 (1.3)	1.2 (1.5)	0.217
Cognition				
SCIP total, (SD)	73.6 (12.5)	77.1 (12.1)	78.2 (12.9)	
TMT-A, (SD)	30.5 (11.5)	29.2 (11.2)	28.8 (8.3)	
TMT-B, (SD)	79.9 (37.6)	78.4 (35.4)	72.3 (22.9)	
Diagnoses				
Bipolar disorder, n (%)	31 (27)	NA	NA	
Unipolar disorder, n (%)	84 (73)	NA	NA	
Age of onset, years (SD)	23.4 (7.7)	NA	NA	
Duration of affective disorder, years (SD)	12.6 (7.6)	NA	NA	
Affective episodes, n (SD)	3.4 (4.8)	NA	NA	
Admissions, n (SD)	2.3 (10.3)	NA	NA	
Months in remission, (SD)	42.7 (50.4)	NA	NA	
Medication				
Current medication, n (%)	73 (63.5)	9 (18.4)	6 (15)	
Antidepressants, n (%)	45 (39.0)	1 (2.0)	0	
Antipsychotics, n (%)	18 (15.7)	0	0	
Mood stabilizers, n (%)	22 (19.1)	0	0	

HDRS-17: Hamilton depression Rating Scale—17 item. YMRS: Young Mania Rating Scale. CI: Confidence interval. SCIP: Screening for Cognitive Impairment in Psychiatry. TMT-A = trail making test, part A, TMT-B= trail making test, part B. Current medication: any current medication. Antidepressive antipsychotics and mood stabilizers were recorded as used at any time point.

**Table 2:** Apolipoprotein E (APOE) genotype frequency distribution across concordance groups: healthy twin pairs, discordant twin pairs and concordant affected monozygotic twins pairs.

		Concordance Groups		
		Low-Risk Twins	Discordant Twins	Concordant Affected Twins
APOE Genotype	E22	0	2	0
		0.0%	2.2%	0,0%
	E32	6	13	11
		15.8%	14.4%	20.8%
	E33	24	43	26
		63.2%	47.8%	49.1%
	E42	2	2	2
		5.3%	2.2%	3.8%
	E43	6	26	8
		15.8%	28.9%	15.1%
	E44	0	4	6
		0.0%	4.4%	11.3%

**Table 3:** Primary, secondary, and tertiary analysis comparing plasma apolipoprotein E (ApoE) levels, triglycerides, high-density lipoprotein (HDL), low-density lipoprotein (LDL), and cholesterol in affected, high-risk, and low-risk monozygotic twins.

3.A: Primary Analyses					Post hoc Group-Wise Comparison, *p*			Post hoc Group-Wise Comparison, Adjusted *p*		
Risk Status	Affected (n = 105)	High Risk (n = 48)	Low Risk (n = 38)	*p*	AF vs. LR	AF vs. HR	HR vs. LR	AF vs. LR	AF vs. HR	HR vs. LR
ApoE Titer (mg/L, CI)	41.9 (39.5–44.4)	38.9 (35.9–41.9)	42.1 (37.3–46.9)	0.097	0.951	0.035	0.261	0.906	0.045	0.345
Triglycerides (mmol/L, CI)	1.1 (1.0–1.2)	0.9 (0.8–1.1)	0.8 (0.5–1.0)	0.052	0.026	0.176	0.189	0.962	0.012	0.048
HDL (nmol/L, CI)	1.6 (1.5–1.7)	1.6 (1.5–1.7)	1.7 (1.5–1.9)	0.289	0.118	0.946	0.148	0.095	0.994	0.116
LDL (nmol/L, CI)	2.9 (2.7–3.1)	2.9 (2.7–3.1)	2.7 (2.4–3.1)	0.731	0.524	0.696	0.440	0.390	0.621	0.300
Cholesterol (nmol/L, CI)	4.8 (4.5–5.0)	4.8 (4.5–5.0)	4.7 (4.2–5.1)	0.888	0.698	0.811	0.630	0.565	0.759	0.487
3.B: Secondary concordance analyses					Post hoc pairwise comparison, *p*			Post hoc adjusted pairwise comparison, *p*		
Risk Status	Concordant affected (25 twin pairs)	Discordant (45 twin pairs)	Low risk (19 twin pairs)	*p*	CA vs. LR	CA vs. Di	Di vs. LR	CA vs. LR	CA vs. Di	Di vs. LR
ApoE Titer (mg/L, CI)	44.0 (39.7–48.2)	38.6 (35.4–41.8)	42.1 (37.2–47.0)	0.260	0.568	0.046	0.231	0.549	0.054	0.282
Triglycerides (mmol/L, CI)	1.3 (1.1–1.5)	0.9 (0.7–1.1)	0.7 (0.5–1.0)	0.025	0.003	0.008	0.362	0.002	0.008	0.264
HDL (nmol/L, CI)	1.7 (1.5–1.7)	1.5 (1.4–1.7)	1.7 (1.5–1.9)	0.439	0.595	0.260	0.107	0.505	0.253	0.086
LDL (nmol/L, CI)	2.7 (2.4–3.1)	2.9 (2.7–3.2)	2.7 (2.4–3.2)	0.764	0.836	0.310	0.485	0.940	0.333	0.353
Cholesterol (nmol/L, CI)	4.6 (4.3–5.0)	4.7 (4.5–5.0)	4.6 (4.3–5.1)	0.876	0.849	0.595	0.785	0.944	0.659	0.644
3.C: Tertiary discordance analyses										
Risk Status	Affected twin (n = 45)	Unaffected twin (n = 45)	*p*							
ApoE Titer (mg/L)	39.4	36.8	0.037							
Triglycerides (mmol/L)	0.9	0.9	0.542							
HDL (nmol/L)	1.5	1.5	0.826							
LDL (nmol/L)	2.8	2.9	0.590							
Cholesterol (nmol/L)	4.6	4.7	0.594							

**Table 4:** Spearman’s correlations between plasma apolipoprotein E (ApoE) levels, lipids, and the cognitive measures across all participants.

	SCIP-VFT	SCIP-VLT-1	SCIP-VLT-D	SCIP-WMT	SCIP-PST	SCIP-total	TMT-A	TMT-B	[ApoE]
[ApoE]									
*p*	0.625	0.604	0.909	0.974	0.696	0.808	0.890	0.642	-
*r*	−0.04	−0.04	−0.01	0.00	−0.03	−0.02	−0.01	0.03	-
[Triglycerides]									
*p*	0.727	0.641	0.499	0.799	0.295	0.342	0.223	0.871	0.000
*r*	−0.02	−0.03	−0.05	−0.02	−0.07	−0.07	0.09	0.01	0.44
[Cholesterol]									
*p*	0.487	0.546	0.601	0.050	0.026	0.114	0.003	0.010	0.000
*r*	−0.05	−0.04	−0.04	−0.14	−0.16	−0.11	0.21	0.18	0.41
[HDL]									
*p*	0.112	0.001	0.018	0.864	0.360	0.022	0.453	0.989	0.996
*r*	0.11	0.24	0.17	−0.01	0.06	0.16	0.05	0.00	0.00
[LDL]									
*p*	0.514	0.099	0.268	0.079	0.044	0.066	0.009	0.016	0.000
*r*	−0.05	−0.12	−0.08	−0.12	−0.14	−0.13	0.18	0.17	0.35

HDL: High-Density Lipoproteins, LDL: Low-Density Lipoproteins. SCIP: Screening for Cognitive Impairment in Psychiatry—Danish version. SCIP-VFT = Verbal Fluency Test, SCIP-VLT-I = Verbal learning test-immediate, SCIP-VLT-D = Verbal learning test-D, SCIP-WMT = Working Memory Test, SCIP-PST= Processing Speed Test, TMT-A = Trail Making Test, part A, TMT-B= Trail Making Test, part B.

## Data Availability

Dataset available on request from the authors.

## References

[1] WHO WHO Fact Sheet for Depresion. https://www.who.int/news-room/fact-sheets/detail/depression.

[2] Global Health Data Exchange (GHDx). http://ghdx.healthdata.org/gbd-results-tool?params=gbd-api-2019-permalink/d780dffbe8a381b25e1416884959e88b.

[3] Craddock N., Sklar P. (2013). Series Bipolar Disorder 1 Genetics of bipolar disorder Search strategy and selection criteria. Lancet.

[4] Johansson V., Kuja-Halkola R., Cannon T.D., Hultman C.M., Hedman A.M. (2019). A population-based heritability estimate of bipolar disorder-In a Swedish twin sample. Psychiatry Res..

[5] Saha S., Lim C.C., Degenhardt L., Cannon D.L., Bremner M., Prentis F., Lawrence Z., Heffernan E., Meurk C., Reilly J. (2022). Comorbidity between mood and substance-related disorders: A systematic review and meta-analysis. Aust. N. Z. J. Psychiatry.

[6] Hafeman D.M., Merranko J., Joseph H.M., Goldstein T.R., Goldstein B.I., Levenson J., Axelson D., Monk K., Sakolsky D., Iyengar S. (2023). Early Indicators of Bipolar Risk in Preschool Offspring of Parents with Bipolar Disorder. J. Child Psychol. Psychiatry.

[7] Jonker I., Rosmalen J.G.M., Schoevers R.A. (2017). Childhood Life Events, Immune Activation and the Development of Mood and Anxiety Disorders: The TRAILS Study. Transl. Psychiatry.

[8] Kühnel A., Czisch M., Sämann P.G., Binder E.B., Kroemer N.B., Brückl T., Spoormaker V.I., Erhardt A., Grandi N.C., Ziebula J. (2022). Spatiotemporal Dynamics of Stress-Induced Network Reconfigurations Reflect Negative Affectivity. Biol. Psychiatry.

[9] Pantazatos S.P., Melhem N.M., Brent D.A., Zanderigo F., Bartlett E.A., Lesanpezeshki M., Burke A., Miller J.M., Mann J.J. (2022). Ventral Prefrontal Serotonin 1A Receptor Binding: A Neural Marker of Vulnerability for Mood Disorder and Suicidal Behavior?. Mol. Psychiatry.

[10] Ceban F., Nogo D., Carvalho I.P., Lee Y., Nasri F., Xiong J., Lui L.M.W., Subramaniapillai M., Gill H., Liu R.N. (2021). Association between Mood Disorders and Risk of COVID-19 Infection, Hospitalization, and Death. JAMA Psychiatry.

[11] Amare A.T., Schubert K.O., Klingler-Hoffmann M., Cohen-Woods S., Baune B.T. (2017). The genetic overlap between mood disorders and cardiometabolic diseases: A systematic review of genome wide and candidate gene studies. Transl. Psychiatry.

[12] Goldstein B.I., Carnethon M.R., Matthews K.A., Mcintyre R.S., Miller G.E., Raghuveer G., Stoney C.M., Wasiak H., Mccrindle B.W. (2015). Major Depressive Disorder and Bipolar Disorder Predispose Youth to Accelerated Atherosclerosis and Early Cardiovascular Disease. Circulation.

[13] Kessing L.V., Ziersen S.C., Andersen P.K., Vinberg M. (2021). A Nation-Wide Population-Based Longitudinal Study Mapping Physical Diseases in Patients with Bipolar Disorder and Their Siblings. J. Affect. Disord..

[14] O’rahilly S. (2009). Human genetics illuminates the paths to metabolic disease. Nature.

[15] Willemsen G., Ward K.J., Bell C.G., Christensen K., Bowden J., Dalgård C., Harris J.R., Kaprio J., Lyle R., Magnusson P.K.E. (2015). The Concordance and Heritability of Type 2 Diabetes in 34,166 Twin Pairs From International Twin Registers: The Discordant Twin (DISCOTWIN) Consortium. Twin Res. Hum. Genet..

[16] Golden S.H. (2008). Examining a Bidirectional Association between Depressive Symptoms and Diabetes. JAMA.

[17] Kemp D.E., Gao K., Chan P.K., Ganocy S.J., Findling R.L., Calabrese J.R. (2010). Medical Comorbidity in Bipolar Disorder: Relationship between Illnesses of the Endocrine/Metabolic System and Treatment Outcome. Bipolar. Disord..

[18] Zhao F., Yue Y., Jiang H., Yuan Y. (2019). Shared genetic risk factors for depression and stroke. Prog. Neuro-Psychopharmacol. Biol. Psychiatry.

[19] Mansur R.B., Brietzke E., McIntyre R.S. (2015). Is There a “Metabolic-Mood Syndrome”? A Review of the Relationship between Obesity and Mood Disorders. Neurosci. Biobehav. Rev..

[20] Atti A.R., Valente S., Iodice A., Caramella I., Ferrari B., Albert U., Mandelli L., De Ronchi D. (2019). Metabolic Syndrome, Mild Cognitive Impairment, and Dementia: A Meta-Analysis of Longitudinal Studies. Am. J. Geriatr. Psychiatry.

[21] Chow Y.Y., Verdonschot M., Mcevoy C.T., Peeters G. (2022). Associations between depression and cognition, mild cognitive impairment and dementia in persons with diabetes mellitus: A systematic review and meta-analysis. Diabetes Res. Clin. Pract..

[22] Diniz B.S., Teixeira A.L., Cao F., Gildengers A., Soares J.C., Butters M.A., Reynolds C.F. (2017). History of Bipolar Disorder and the Risk of Dementia: A Systematic Review and Meta-Analysis. Am. J. Geriatr. Psychiatry.

[23] Rock P.L., Roiser J.P., Riedel W.J., Blackwell A.D. (2014). Cognitive Impairment in Depression: A Systematic Review and Meta-Analysis. Psychol. Med..

[24] Scult M.A., Paulli A.R., Mazure E.S., Moffitt T.E., Hariri A.R., Strauman T.J. (2016). The Association between Cognitive Function and Subsequent Depression: A Systematic Review and Meta-Analysis. Psychol. Med..

[25] Szmulewicz A., Valerio M.P., Martino D.J. (2020). Longitudinal Analysis of Cognitive Performances in Recent-Onset and Late-Life Bipolar Disorder: A Systematic Review and Meta-Analysis. Bipolar. Disord..

[26] Varghese S., Frey B.N., Schneider M.A., Kapczinski F., de Azevedo Cardoso T. (2022). Functional and Cognitive Impairment in the First Episode of Depression: A Systematic Review. Acta Psychiatr. Scand..

[27] Du H., Meng X., Yao Y., Xu J. (2022). The Mechanism and Efficacy of GLP-1 Receptor Agonists in the Treatment of Alzheimer’s Disease. Front. Endocrinol. (Lausanne).

[28] Elliott D.A., Weickert C.S., Garner B. (2010). Apolipoproteins in the Brain: Implications for Neurological and Psychiatric Disorders. Clin. Lipidol..

[29] Jha A., Lammertse D.P., Coll J.R., Charlifue S., Coughlin C.T., Whiteneck G.G., Worley G. (2016). Apolipoprotein E S4 Allele and Outcomes of Traumatic Spinal Cord Injury. J. Spinal Cord. Med..

[30] Knöchel C., Kniep J., Cooper J.D., Stäblein M., Wenzler S., Sarlon J., Prvulovic D., Linden D.E.J., Bahn S., Stocki P. (2017). Altered Apolipoprotein C Expression in Association with Cognition Impairments and Hippocampus Volume in Schizophrenia and Bipolar Disorder. Eur. Arch. Psychiatry Clin. Neurosci..

[31] Van Reedt Dortland A.K.B., Giltay E.J., van Veen T., van Pelt J., Zitman F.G., Penninx B.W.J.H. (2010). Associations between Serum Lipids and Major Depressive Disorder. J. Clin. Psychiatry.

[32] Ebrahim I.M., Ghahremani M., Camicioli R., Smith E.E., Ismail Z. (2023). Effects of Race, Baseline Cognition, and APOE on the Association of Affective Dysregulation with Incident Dementia: A Longitudinal Study of Dementia-Free Older Adults. J. Affect. Disord..

[33] Kessing L.V., Jørgensen O.S. (1999). Apolipoprotein E-Ε4 Frequency in Affective Disorder. Biol. Psychiatry.

[34] López-León S., Janssens A.C.J.W., González-Zuloeta Ladd A.M., Del-Favero J., Claes S.J., Oostra B.A., van Duijn C.M. (2008). Meta-Analyses of Genetic Studies on Major Depressive Disorder. Mol. Psychiatry.

[35] Gatt J.M., Burton K.L.O., Williams L.M., Schofield P.R. (2014). Specific and common genes implicated across major mental disorders: A review of meta-analysis studies. J. Psychiatr. Res..

[36] Rasmussen K.L., Tybjærg-Hansen A., Nordestgaard B.G., Frikke-Schmidt R. (2019). Plasma Levels of Apolipoprotein E, APOE Genotype, and All-Cause and Cause-Specific Mortality in 105 949 Individuals from a White General Population Cohort. Eur. Heart J..

[37] Rasmussen K.L., Tybjærg-Hansen A., Nordestgaard B.G., Frikke-Schmidt R. (2016). Plasma Levels of Apolipoprotein E and Risk of Ischemic Heart Disease in the General Population. Atherosclerosis.

[38] Dean B., Digney A., Sundram S., Thomas E., Scarr E. (2008). Plasma Apolipoprotein E Is Decreased in Schizophrenia Spectrum and Bipolar Disorder. Psychiatry Res..

[39] Sadeghi M., Roohafza H., Afshar H., Rajabi F., Ramzani M., Shemirani H., Sarafzadeghan N. (2011). Relationship between Depression and Apolipoproteins A and B: A Case-Control Study. Clinics.

[40] Ottesen N.M., Meluken I., Frikke-Schmidt R., Plomgaard P., Scheike T., Fernandes B.S., Berk M., Poulsen H.E., Kessing L.V., Miskowiak K. (2020). Are Remitted Affective Disorders and Familial Risk of Affective Disorders Associated with Metabolic Syndrome, Inflammation and Oxidative Stress?—A Monozygotic Twin Study. Psychol. Med..

[41] Ottesen N.M., Meluken I., Scheike T., Kessing L.V., Miskowiak K.W., Vinberg M. (2018). Clinical Characteristics, Life Adversities and Personality Traits in Monozygotic Twins with, at Risk of and without Affective Disorders. Front. Psychiatry.

[42] Wing J.K., Babor T., Brugha T., Burke J., Cooper J.E., Giel R., Jablenski A., Regier D., Sartorius N. (1990). SCAN. Schedules for Clinical Assessment in Neuropsychiatry. Arch. Gen. Psychiatry.

[43] Hamilton M. (1967). Development of a Rating Scale for Primary Depressive Illness. Br. J. Soc. Clin. Psychol..

[44] Young R.C., Biggs J.T., Ziegler V.E., Meyer D.A. (1978). A Rating Scale for Mania: Reliability, Validity and Sensitivity. Br. J. Psychiatry.

[45] Bowie C.R., Harvey P.D. (2006). Administration and interpretation of the Trail Making Test. Nat. Protoc..

[46] Jensen J.H., Støttrup M.M., Nayberg E., Knorr U., Ullum H., Purdon S.E., Kessing L.V., Miskowiak K.W. (2015). Optimising Screening for Cognitive Dysfunction in Bipolar Disorder: Validation and Evaluation of Objective and Subjective Tools. J. Affect. Disord..

[47] Ott C.V., Bjertrup A.J., Jensen J.H., Ullum H., Sjælland R., Purdon S.E., Vieta E., Kessing L.V., Miskowiak K.W. (2016). Screening for Cognitive Dysfunction in Unipolar Depression: Validation and Evaluation of Objective and Subjective Tools. J. Affect. Disord..

[48] Jordan B.D. (2007). Genetic Influences on Outcome Following Traumatic Brain Injury. Neurochem. Res..

[49] Feng F., Lu S.S., Hu C.Y., Gong F.F., Qian Z.Z., Yang H.Y., Wu Y.L., Zhao Y.Y., Bi P., Sun Y.H. (2015). Association between Apolipoprotein e Gene Polymorphism and Depression. J. Clin. Neurosci..

[50] De Souza M.G.S., Bio D.S., Dias V.V., do Prado C.M., Campos R.N., Costa L.F.d.O., Moreno D.H., Ojopi E.B., Gattaz W.F., Moreno R.A. (2010). Short Communication: Apolipoprotein E Genotype and Cognition in Bipolar Disorder. CNS Neurosci. Ther..

[51] Meller M.R., Patel S., Duarte D. (2021). Kapczinski, | Flavio Bipolar Disorder and Frontotemporal Dementia: A Systematic Review. Acta Psychiatr. Scand..

[52] Rubin R. (2018). Exploring the Relationship between Depression and Dementia. JAMA.

[53] Rasmussen K.L., Tybjærg-Hansen A., Nordestgaard B.G., Frikke-Schmidt R. (2018). Plasma Apolipoprotein E Levels and Risk of Dementia: A Mendelian Randomization Study of 106,562 Individuals. Alzheimer’s Dementia.

[54] Nordestgaard L.T., Christoffersen M., Afzal S., Nordestgaard B.G., Tybjærg-Hansen A., Frikke-Schmidt R. (2021). Triglycerides as a Shared Risk Factor between Dementia and Atherosclerotic Cardiovascular Disease: A Study of 125 727 Individuals. Clin. Chem..

[55] Van Rheenen T.E., Mcintyre R.S., Balanzá-martínez V., Berk M., Rossell S.L. (2021). Cumulative Cardiovascular Disease Risk and Triglycerides Differentially Relate to Subdomains of Executive Function in Bipolar Disorder; Preliminary Findings. J. Affect. Disord..

[56] Wei Y., Cai D., Liu J., Liu R., Wang S. (2020). Cholesterol and Triglyceride Levels in First-Episode Patients with Major Depressive Disorder: A Meta-Analysis of Case-Control Studies. J. Affect. Disord..

[57] Lane-Donovan C., Wong W.M., Durakoglugil M.S., Wasser C.R., Jiang S., Xian X., Herz J. (2016). Genetic Restoration of Plasma Apoe Improves Cognition and Partially Restores Synaptic Defects in ApoE-Deficient Mice. J. Neurosci..

[58] Mooijaart S.P., Van Vliet P., Van Heemst D., Rensen P.C.N., Berbéeberb´berbée J.F.P., Jolles J., De Craen A.J.M., Westendorp R.G.J. (2007). Plasma Levels of Apolipoprotein E and Cognitive Function in Old Age. Ann. N. Y. Acad. Sci.

[59] Song F., Poljak A., Crawford J., Kochan N.A., Wen W. (2012). Plasma Apolipoprotein Levels Are Associated with Cognitive Status and Decline in a Community Cohort of Older Individuals. PLoS ONE.

[60] Morley J.E., Banks W.A. (2010). Lipids and Cognition. J. Alzheimer’s Dis..

[61] Shao T.N., Yin G.Z., Yin X.L., Wu J.Q., Du X.D., Zhu H.L., Liu J.H., Wang X.Q., Xu D.W., Tang W.J. (2017). Elevated Triglyceride Levels Are Associated with Cognitive Impairments among Patients with Major Depressive Disorder. Compr. Psychiatry.

[62] Vicent Sánchez-Ortí J., Balanzá-Martínez V., Correa-Ghisays P., Selva-Vera G., Vila-Francés J., Magdalena-Benedito R., Escribano-Lopez I., Crespo-Facorro B., Tabarés-Seisdedos R. (2022). Specific Metabolic Syndrome Components Predict Cognition and Social Functioning in People with Type 2 Diabetes Mellitus and Severe Mental Disorders. Acta Psychiatr. Scand..

[63] Anstey K.J., Lipnicki D.M., Low L.F. (2008). Cholesterol as a risk factor for dementia and cognitive decline: A systematic review of prospective studies with meta-analysis. Am. J. Geriatr. Psychiatry.

[64] Hua R., Ma Y., Li C., Zhong B., Xie W. (2021). Low Levels of Low-Density Lipoprotein Cholesterol and Cognitive Decline. Sci. Bull..

[65] Penninx B.W.J.H., Lange S.M.M. (2022). Metabolic Syndrome in Psychiatric Patients: Overview, Mechanisms, and Implications. Dialogues Clin. Neurosci..

[66] Christensen K., Petersen I., Skytthe A., Herskind A.M., McGue M., Bingley P. (2006). Comparison of academic performance of twins and singletons in adolescence: Follow-up study. Br. Med. J..

[67] Kläning U., Laursen T.M., Licht R.W., Kyvik K.O., Skytthe A., Mortensen P.B. (2004). Is the Risk of Bipolar Disorder in Twins Equal to the Risk in Singletons? A Nationwide Register-Based Study. J. Affect. Disord..

[68] Öberg S., Cnattingius S., Sandin S., Lichtenstein P., Morley R., Iliadou A.N. (2012). Twinship Influence on Morbidity and Mortality across the Lifespan. Int. J. Epidemiol..

[69] Petersen I., Nielsen M.M.F., Beck-Nielsen H., Christensen K. (2011). No Evidence of a Higher 10 Year Period Prevalence of Diabetes among 77,885 Twins Compared with 215,264 Singletons from the Danish Birth Cohorts 1910–1989. Diabetologia.

[70] Bora E. (2018). Neurocognitive Features in Clinical Subgroups of Bipolar Disorder: A Meta-Analysis. J. Affect. Disord..

[71] Chan J.K.N., Tong C.H.Y., Wong C.S.M., Chen E.Y.H., Chang W.C. (2022). Life Expectancy and Years of Potential Life Lost in Bipolar Disorder: Systematic Review and Meta-Analysis. Br. J. Psychiatry.

[72] Erlangsen A., Andersen P.K., Toender A., Laursen T.M., Nordentoft M., Canudas-Romo V. (2017). Cause-Specific Life-Years Lost in People with Mental Disorders: A Nationwide, Register-Based Cohort Study. Lancet Psychiatry.

[73] Kessing L.V., Vradi E., Andersen P.K. (2015). Life Expectancy in Bipolar Disorder. Bipolar. Disord..

[74] Goldfarb M., De Hert M., Detraux J., Di Palo K., Munir H., Music S., Piña I., Ringen P.A. (2022). Severe Mental Illness and Cardiovascular Disease. J. Am. Coll. Cardiol..

[75] Momen N.C., Plana-Ripoll O., Agerbo E., Christensen M.K., Iburg K.M., Laursen T.M., Mortensen P.B., Pedersen C.B., Prior A., Weye N. (2022). Mortality Associated with Mental Disorders and Comorbid General Medical Conditions. JAMA Psychiatry.

[76] Liu Y.K., Ling S., Lui L.M.W., Ceban F., Vinberg M., Kessing L.V., Ho R.C., Rhee T.G., Gill H., Cao B. (2022). Prevalence of Type 2 Diabetes Mellitus, Impaired Fasting Glucose, General Obesity, and Abdominal Obesity in Patients with Bipolar Disorder: A Systematic Review and Meta-Analysis. J. Affect. Disord..

